# Natural Is Not Always Better: The Varied Effects of a Natural Environment and Exercise on Affect and Cognition

**DOI:** 10.3389/fpsyg.2020.575245

**Published:** 2021-01-11

**Authors:** Janet P. Trammell, Shaya C. Aguilar

**Affiliations:** Social Science Department, Seaver College, Pepperdine University, Malibu, CA, United States

**Keywords:** Attention Restoration Theory, affect (emotion, mood, personality), attention, cognition, natural environment, exercise

## Abstract

The Attention Restoration Theory (ART) has been widely cited to account for beneficial effects of natural environments on affect and attention. However, the effects of environment and exercise are not consistent. In a within-subjects design, participants completed affective and cognitive measures that varied in attentional demands (memory, working memory, and executive function) both before and after exercise in a natural and indoor environment. Contrary to the hypotheses, a natural environment resulted in lower positive affect and no difference in negative affect compared to an indoor environment. A natural environment resulted in the most improvement for cognitive tasks that required moderate attentional demand: Trail Making Test A and Digit Span Forwards. As predicted, exercise resulted in improved affect and improved executive function (Trail Making Test B). There were no interactions between environment and exercise. These results suggest that ART cannot fully explain the influence of environment on affect and cognition.

## Introduction

Nature is more than a physical environment; it is an environment that can both restore and enhance the mind and behavior. It is well established that time spent in natural environments is associated with beneficial outcomes for mental health, such as increases in positive affect and decreases in stress, negative affect, anger, fatigue, and sadness (see Bowler et al., 2010, and McMahan and Estes, 2015, for a review of affective benefits; but see Gascon et al., 2015 for limitations). These natural environments typically contain green and/or blue spaces; green spaces are spacious, lush, serene, and include vegetation such as trees, grass, forests, and parks, whereas blue spaces include all kinds of water such as lakes, rivers, and the ocean (Gascon et al., 2015). People are more likely to be physically active as well as experience feelings of peace and restoration in green and blue spaces (Finlay et al., 2015), with the largest benefit in positive affect resulting from areas that have a combination of both green and blue spaces (White et al., 2010).

In addition to affective benefits, natural environments are also associated with cognitive benefits, particularly in regards to attention. According to Attention Restoration Theory (ART; Kaplan, 1995; Berman et al., 2008; Kaplan and Berman, 2010), natural environments capture attention in an involuntary but undemanding way. This “soft fascination” is in contrast to the direct and focused attentional demands of most other environments, and results in improved affect as well as allowing attentional resources—particularly those involved in focused directed attention, which is utilized in cognitively demanding tasks—a chance to recover. Stress reduction theory (SRT; Ulrich et al., 1991) posits that rather than soft fascination leading to both attentional restoration and affective benefits, the affective benefits resulting from exposure to natural environments lead to attentional restoration. However, recent data suggests that affective benefits are not the cause for cognitive benefits; rather, the two are more independent (Schertz and Berman, 2019).

Compared to exposure to urban environments, many studies have found attentional improvements as a result of exposure to natural environments. These improvements occur across young and old adults (Gamble et al., 2014), mentally fatigued individuals (Berto, 2005), and children with ADHD (Taylor and Kuo, 2009). Lending further support, two meta-analyses (Ohly et al., 2016; Stevenson et al., 2018) reported that performance on tasks that utilize both directed attention and working memory [Digit Span Forwards (DSF)/Digit Span Backwards (DSB)] and both directed visual attention and executive function [sometimes called cognitive flexibility; Trail Making Test B (TMTB)] is improved as a result of exposure to natural environments. However, these cognitive benefits are less consistent than affective benefits; no consistent improvement was found for Trail Making Test A (TMTA; Ohly et al., 2016), vigilance, impulse control, or processing speed (Stevenson et al., 2018), and an earlier meta-analysis did not find support for improvement in attention after exposure to a natural environment (Bowler et al., 2010).

Why might cognitive benefits be inconsistent? The demands of the task likely matter (Ohly et al., 2016; Stevenson et al., 2018), such that more demanding tasks (DSB, TMTB) are more likely to show benefits of nature-based restoration than less demanding tasks (DSF, TMTA). It is also likely that participants’ degree of fatigue or need for restoration influences the amount of restoration experienced (Stevenson et al., 2018). In addition, while ART focuses primarily on attentional restoration, tasks such as Digit Span and Trail Making also rely heavily on other cognitive resources, such as working memory and executive function, the effect of environment on which is less understood.

Importantly, while effects on attention have been extensively investigated, the effect of environment on tasks with low attentional demands, such as long-term memory, has rarely been investigated. In the only experimental investigation to date of the effects of a natural environment (a 10 min walk) on memory, no significant effects of environment were found (Rider and Bodner, 2016); however, actual encoding and recall took place in an indoor environment, not in the natural or urban walk environments. If improvement in performance on tasks such as Digit Span and Trail Making is due purely to attentional restoration, as proposed in ART, then we would not expect to see environmental differences on a memory task. If, however, improvements in performance are due to other factors, such as increased interest and motivation (Joye and Dewitte, 2018), or affective benefits leading to cognitive benefits (SRT; Ulrich et al., 1991), then we should see similar improvements in memory as for tasks with greater attentional demand.

A second consideration is that much of this research has focused on simple exposure to natural environments—such as viewing pictures or videos (e.g., Gamble et al., 2014), or exercising at light intensity by walking (e.g., Gidlow et al., 2016). It is possible that with more activity, such as moderate intensity exercise, the environment would have different effects, particularly as moderate exercise is presumed to physically fatigue individuals regardless of the environment in which it takes place. Additionally, while indoor environments have been somewhat investigated, most comparisons to a natural environment are an urban environment; however, for the majority of adults who work, learn, or otherwise engage in cognitively demanding tasks, they must do so indoors rather than in an outdoor urban environment.

Exercise, of course, has a separate influence on affect and cognition. Similar to natural environments, it is well established that physical exercise, regardless of whether it is an acute episode or long term engagement, has numerous physical and mental health benefits. For instance, exercise has a therapeutic and occasionally protective effect on mood and cognition for those diagnosed with ADHD and autism (Tan et al., 2016), Alzheimer’s (Farina et al., 2014), Mild Cognitive Impairment (Öhman et al., 2014), and other conditions. In particular, “green exercise,” or exercise in green outdoor spaces, has been shown to have benefits such as increased energy, engagement and revitalization, and decreased depression and tension (for reviews, see Pretty et al., 2007; Coon et al., 2011).

Over the last several decades, cognitive benefits of exercise, in various domains such as attention, memory, learning, speed, processing, and executive function, have been robustly demonstrated across the lifespan in children (Tomporowski et al., 2011), young-to-middle age adults (Hötting et al., 2012; Loprinzi et al., 2018), and older adults (Colcombe and Kramer, 2003). The intensity and duration of exercise are important factors influencing these effects. Acute aerobic exercise of at least 20 min produces stronger effects on cognition than shorter bouts, particularly when cognitive assessments are completed 11–20 min after the cessation of exercise (Chang et al., 2012). Regarding intensity, evidence suggests that moderate intensity leads to larger benefits for cognition than lower intensity (e.g., Naderi et al., 2019), and that exercise of greater intensity may produce longer-lasting benefits (Chang et al., 2012).

Despite these robust and generally consistent findings, however, there are still unanswered questions concerning the interplay between exercise and cognition. First, benefits on cognition are not always consistent; while both Chang et al. (2012) and Lambourne and Tomporowski (2010) find strong meta-analytic support for beneficial effects of acute exercise on long term memory, effects on other cognitive functions, such as working memory and executive function, are less consistent. This may be because effect sizes are typically larger for long term memory effects than for executive function (Lambourne and Tomporowski, 2010). Further, particularly in regards to working memory, the duration and intensity of exercise, and the particular measures used, contribute to the variances in the findings. Second, research has typically focused on exercise conducted in a laboratory, gym, or otherwise generally static, typically indoor environment. However, a laboratory is not a typical exercise environment; many adults lack access to a fitness facility and thus exercise outdoors by running, cycling, walking, gardening, and hiking, etc. According to ART, there is reason to think that an exercise environment may be an important factor in effects on cognition, with natural environments being more beneficial. Research into the cognitive benefits of “green exercise” has been steadily increasing, albeit with inconsistent results and typically focusing primarily on attentional tasks. One study utilizing running (Bodin and Hartig, 2003) found no attentional benefits for a natural compared to an urban environment, although statistical power was low. Others (Rogerson and Barton, 2015) have found that viewing a nature video during indoor treadmill running resulted in greater improvement on DSB scores than viewing an urban video or no video.

While key assumptions of ART are the ideas that directed attention can be depleted, and that natural environments restore this resource, the conceptualization of “directed attention” is not clearly defined (Ohly et al., 2016; Joye and Dewitte, 2018). The mechanism for improved cognitive performance after exposure to natural environments could be affective benefits leading to increased motivation and persistence on cognitively demanding tasks rather than restoration of a depleted resource (Joye and Dewitte, 2018). In order to more effectively test the attention-restoring claims of ART, Joye and Dewitte (2018) make several recommendations, two of which we will undertake here: (1) including a non-fatigued control group (here, affective and cognitive assessment at the beginning of exposure to each environment) and (2) using within-subjects comparisons to assess identical pre- and post- environment measures. These recommendations help to determine the limits of restoration: when participants are not yet fatigued at the first measurement, will being in a natural environment benefit affect and cognition? Or will any benefits of a natural environment be evident only after participants experience fatigue (induced through the first set of cognitive tests and exercise)?

By testing the effect of environment and exercise on affect and cognition in Experiment 1, and further exploration of the restorative characteristics of the natural environment in Experiment 2, we aim to: (1) more effectively test the attention restoration claims of ART as suggested by Joye and Dewitte (2018) and (2) to provide clarity for the effects of environment and inconsistent effects of exercise on different cognitive tasks varying in attentional demands, such as short term recall and long term recognition memory (little attentional demand), working memory (DSF: moderate attentional demand/DSB: greater attentional demand), and executive function (TMTA: moderate attentional demand; TMTB: greater attentional demand). Accordingly, we compared affect and the cognitive performance of adults both before and after both an outdoor trail run in a natural environment and an indoor treadmill run in a laboratory environment. If ART theory is supported, affect and tasks involving attention (DSF/DSB, TMTA/TMTB) would be improved in the natural environment condition compared to the indoor condition, with the largest benefits in the tests that require the greatest attentional demands, and no benefit expected for memory. If instead other factors, such as interest, motivation, or stress reduction are driving performance differences, then all cognitive measures should show improvement in the natural environment compared to the indoor environment, regardless of attentional demands. Further, if ART theory is supported, we expect an interaction, such that non-fatigued participants at the beginning of exposure to the environment should not differ in affective and cognitive measures as a function of their environment, but that post-exercise restoration would be greater in the natural environment, leading to lower negative affect, greater positive affect, and improved cognitive performance. In regards to exercise, we hypothesized that affect and long term memory would improve after exercise, and that working memory and executive function may differ (e.g., Lambourne and Tomporowski, 2010; Chang et al., 2012).

## Materials and Methods

### Participants

Twenty-eight (13 men, 15 women) regular runners (defined as running at least 1 time per week for at least 6 months) between the ages of 18 and 50 (*M* = 26.96, *SD* = 10.16) were recruited from the University and local population to participate in this 2 (Environment: Natural vs Indoor) X 2 (Exercise: Pre vs Post) within-subjects design. Participants with physical (injury), mental (cognitive impairment), or pharmacological (stimulant medications) indicators were excluded from participation.

### Materials and Measures

#### Exercise Environment

The natural environment was the main hiking and running trail in Solstice Canyon, located in the Santa Monica Mountains National Recreation Area. At the parking area near the trailhead, a shaded pavilion with picnic tables served as the location for completing all pre- and post-exercise measures. The trail (see section “Appendix”) was a wide dirt and gravel-packed path with a gradual slope that followed a stream through trees, a canyon, and historical ruins. At the end of the main trail, a waterfall cascaded into the stream. Participants were instructed to remain on the main trail at all times and to turn around after either slightly more than halfway through the 20 min time period (10 min and 20 s to account for the uphill on the way to the waterfall and downhill on the return) or after reaching the waterfall. If they returned to the beginning of the trailhead before the 20 min was completed, participants were to run the beginning part of the trail again as needed to make their run end at the trailhead at approximately 20 min.

The indoor location consisted of a research laboratory room on the campus of Pepperdine University. The room was divided with a treadmill (Nordic Track T 6.5S) behind a partial wall and table and chairs to complete all affective and cognitive measures on the other side of the wall. To approximate the trail run, participants were instructed to increase to a gradual incline in the first 10 min, and to decrease the incline back to zero during the last 10 min.

#### Exercise Materials

All participants were fitted with a Garmin Fenix 5 Plus watch before each run. A beep sounded to alert the participant to turn around and return to the trailhead after 10 min and 20 s in the natural environment condition. Further, as a precaution in the unlikely event that participants disregarded instructions to remain on the main trailhead or became lost (none did so), participants were shown how to access the map feature on the watch, which provided an option for GPS assisted routing back to the trailhead. The watch also recorded the total running distance and time, which was used to confirm that participants ran with minimal to no walking (i.e., a pace faster than 13 min per mile) and did not run more than 30 s longer or shorter than the instructed 20 min. For the indoor condition, distance, time, and pace was confirmed through the treadmill display. All participants viewed and verbally indicated understanding the Rating of Perceived Exertion (Borg, 1982), a 15 point (6 = *No Exertion at All*, 20 = *Maximal Exertion)* perceived exertion scale prior to each run.

#### Affective Measures

Participants completed the Positive and Negative Affect Schedule Short Form (PANAS-SF; Watson et al., 1988). Participants rated how strongly they were currently experiencing 10 positive (e.g., enthusiastic) and 10 negative (e.g., irritable) feelings on a Likert-scale ranging from 1 (*very slightly or not at all*) to 5 (*extremely*). Total scores could range from 10–50. The PANAS-SF has demonstrated acceptable reliability and validity (Watson et al., 1988). Participants answered two additional items, “happy” and “stressed,” on the same scale.

#### Cognitive Measures

In order to reduce practice effects, all participants completed 4 different versions of each cognitive test: pre- and post-exercise in both the indoor and natural environment.

##### Memory

Participants completed a shortened variant of the Rey Auditory Verbal Learning Task (AVLT; Rey, 1964). The researcher read out loud to participants one of 4 different versions of a 15 item word list. Words were read at a rate of 1 s per word. For short-term recall, immediately after hearing the list, participants reported all of the words they could remember. For long term recognition memory, after a delay of 15 min (during which participants engaged in the two other cognitive tasks, followed by light stretching) the researcher read a list consisting of the previously read 15 words intermixed with 15 new words in random order. After hearing each word, participants indicated which words were on the original list by saying “yes” to old items and “no” to new items. For recognition, the ability to discriminate between “old” and “new” was measured by d’ (*z* of Hit Rate – *z* of False Alarm rate). Hit rate was calculated by the proportion of old items correctly identified as old, and False Alarm rate was calculated by the proportion of new items incorrectly identified as old. All lists contained nouns that were equated for word frequency; the 4 different versions of the recall list were lists 1–4 and the additional 15 new items added to each recognition test were lists 5–8 from Potter and Keeling (2005).

##### Working memory

Participants completed DSF and DSB tests. For DSF, participants heard digit sequences and were required to repeat them in order. Sequences were two to nine digits in length (two sequences of each length for a total of 16 sequences) and were presented in increasing length. For DSB, participants heard digit sequences and were required to repeat them in backwards order (i.e., if the sequence was “3, 2” they were to report “2, 3”). There were 14 sequences, consisting of two sequences each of two to eight digits. For both DSF and DSB, after making mistakes on two sequences or upon completion of the final sequence the task was ended. The number of correct sequences was recorded, with a maximum score of 16 (DSF) or 14 (DSB). In addition, the length of the longest sequence (i.e., span length) recalled correctly was recorded. The total number correct and span length were multiplied to create a product score (Kessels et al., 2000). Four different versions of the DSF and DSB were created and administered.

##### Executive function

Participants completed the Trail Making Test A and B (Bowie and Harvey, 2006). Both parts consisted of 25 circles distributed over a sheet of paper. In TMTA, the circles were numbered 1–25, and the participant drew lines to connect the numbers in ascending order as quickly as possible, without lifting the pen or pencil from the paper. In TMTB, the circles included both numbers (1–13) and letters (A – L); as in TMTA, the participant drew lines to connect the circles in an ascending pattern, but with the added task of alternating between the numbers and letters (i.e., 1-A-2-B-3-C, etc.). If the participant made an error, the researcher pointed it out immediately and instructed him or her to correct it. The total time to complete the test was measured. In addition to the original A and B (Bowie and Harvey, 2006), 3 additional versions were created by rearranging the numbers and letters in each circle.

### Procedure

This research was approved by the Institutional Review Board of Pepperdine University. Interested participants who passed the physical, cognitive, and pharmacological screening completed 2 conditions in random order: a 20-min outdoor trail run (natural environment) and a 20-min treadmill run (indoor environment). Outdoor runs did not take place in hazardous or adverse weather conditions (for example, temperatures above 90 °F/32.2°C or strong winds) or non-daylight hours. Each participant completed their indoor and outdoor runs at approximately the same time of day, 1 week apart.

**FIGURE 1:**
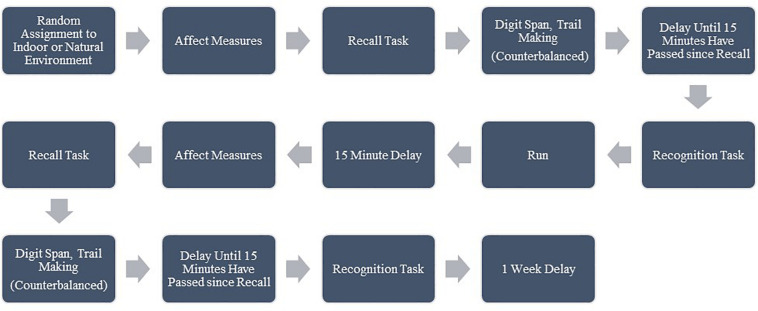
Session 1 procedure; Session 2 followed the same procedure in the alternate environment. Affect Measures included ratings of: happiness, stress, Positive Affect (PA), and Negative Affect (NA). Cognitive measures included a recall and recognition test for word lists, Digit Span Forwards (DSF), Digit Span Backwards (DSB), Trail Making Test A (TMTA), and Trail Making Test (B).

At Session 1, participants met the researcher at either the natural environment (Solstice Canyon main parking area) or the indoor environment (the research room on the University’s campus), depending on which location they were randomly assigned to first. Participants gave informed consent and then completed the first PANAS, happiness, and stress questionnaire followed by the recall portion of the AVLT. Next, participants completed either the Digit Span or Trail Making tests (in random order), followed by light stretching, until 15 min had passed since the completion of the recall test. Then, participants completed the recognition portion of the AVLT. Before their run, participants viewed the perceived exertion scale (Borg, 1982) and received instructions to maintain a moderate to high intensity, or a 14–15. Participants then completed the 20 min run as described above. After the run, participants were offered bottled water and were instructed to engage in light stretching or to alternate walking with sitting for 15 min. Finally, participants completed the second set of affect and cognitive measures, in the same manner as they completed the first set. They were then reminded of the location and time for Session 2 and offered an energy sports snack (such as Clif Blocks, GU Gel, or similar product). Please see Figure 1 for a representation of the procedure.

At Session 2, 1 week after Session 1, participants followed the same procedure as in Session 1 in the other environment with the two remaining versions of the cognitive tasks. They were then debriefed and thanked with a $50 Amazon gift card for their participation.

## Results

With one exception, there were no environment order effects; for the fourth administration (the end of Session 2) of the TMTB, those who had completed their first session indoors (*M* = 31.22, *SD* = 9.24) responded faster than those who had completed their first session in the natural environment (*M* = 45.87, *SD* = 13.33), *F* (1, 27) = 11.51, *p* = 0.002, and η^2^ = 0.31. Given the lack of order effects on any other measure, order was not considered as a factor in further analyses. Similarly, there were no significant gender effects or interactions. For all analyses, a 2 (Environment: Indoor, Natural) × 2 (Exercise: Pre, Post) Repeated Measures ANOVA was conducted to determine the effects of environment and exercise on affect and cognition (with the exception of Trail Making, as described below).

### Affect

#### Happiness

There was a significant effect of environment on happiness, *F* (1, 27) = 5.87, *p* = 0.02, and η_*p*_^2^ = 0.18, such that participants reported more happiness in the indoor environment than in the natural environment, see Table 1. There was also a significant main effect of exercise on happiness, *F* (1, 27) = 5.64, *p* = 0.03, η_*p*_^2^ = 0.17, such that participants were happier after exercise than they were before exercise. The interaction between environment and exercise was not significant, *F* (1, 27) = 0.04, *p* = 0.85, η_*p*_^2^ = 0.001.

**TABLE 1:** Mean (*SD*) affect as a function of location and exercise.

	**Happiness**	**Stress**	**Positive Affect**	**Negative Affect**
Natural environment	3.50 (0.95)	2.11 (1.12)	30.88 (8.63)	12.73 (3.41)
Pre-Exercise	3.39 (0.96)	2.43 (1.17)	28.43 (7.49)	12.93 (3.63)
Post-Exercise	3.61 (0.96)	1.79 (0.99)	33.32 (9.11)	12.54 (3.22)
Indoor environment	3.77 (0.93)	2.07 (1.08)	34.30 (7.41)	13.30 (4.39)
Pre-Exercise	3.68 (0.90)	2.29 (1.24)	32.43 (6.65)	14.18 (5.25)
Post-Exercise	3.86 (0.97)	1.86 (0.85)	36.18 (7.76)	12.43 (3.17)
Pre-Exercise	3.54 (0.93)	2.36 (1.20)	30.43 (7.30)	13.55 (4.51)
Post-Exercise	3.73 (0.96)	1.82 (0.92)	34.75 (8.51)	12.48 (3.17)
Total	3.63 (0.95)	2.09 (1.10)	32.59 (8.19)	13.02 (3.92)

#### Stress

There was no significant effect of environment on stress, *F* (1, 27) = 0.08, *p* = 0.78, and η_*p*_^2^ = 0.003. There was a significant main effect of exercise on stress, *F* (1, 27) = 14.03, *p* = 0.001, and η_*p*_^2^ = 0.34, such that participants were less stressed after exercise than they were before exercise, see Table 1. The interaction between environment and exercise was not significant, *F* (1, 27) = 1.21, *p* = 0.28, and η_*p*_^2^ = 0.04.

#### PANAS

There was a significant effect of environment on positive affect, *F* (1, 27) = 15.72, *p* < 0.001, and η_*p*_^2^ = 0.37, such that participants reported higher positive affect in the indoor environment (than in the natural environment. There was also a significant main effect of exercise on positive affect, *F* (1, 27) = 32.19, *p* < 0.001, and η_*p*_^2^ = 0.54, such that participants reported higher positive affect after exercise than before exercise. The interaction between environment and exercise was not significant, *F* (1, 27) = 1.55, *p* = 0.22, and η_*p*_^2^ = 0.05, see Table 1.

There was no significant effect of environment on negative affect, *F* (1, 27) = 1.60, *p* = 0.22, and η_*p*_^2^ = 0.06. There was a significant main effect of exercise on negative affect, *F* (1, 27) = 4.48, *p* = 0.04, η_*p*_^2^ = 0.14, such that participants reported lower negative affect after exercise than before exercise, see Table 1. The interaction between environment and exercise was marginally significant, *F* (1, 27) = 3.34, *p* = 0.08, and η_*p*_^2^ = 0.11, suggesting that negative affect decreased more after exercise in the indoor condition than in the outdoor condition, see Table 1.

### Cognitive Performance

#### Memory

There was no significant effect of environment [*F* (1, 27) = 0.00, *p* = 1.00, and η_*p*_^2^ = 0.00], exercise [*F* (1, 27) = 1.44, *p* = 0.24, and η_*p*_^2^ = 0.05], or an interaction [*F* (1, 27) = 2.22, *p* = 0.15, and η_*p*_^2^ = 0.08] on correct recall of the word lists. There was a marginal effect of exercise, *F* (1, 27) = 3.14, *p* = 0.09, η_*p*_^2^ = 0.10, such that discrimination (d’) was higher before exercise than after exercise. There was no significant effect of environment [*F* (1, 27) = 0.00, *p* = 0.99, η_*p*_^2^ = 0.00) and no interaction [*F* (1, 27) = 0.14, *p* = 0.72, and η_*p*_^2^ = 0.01), see Table 2.

**TABLE 2:** Mean (*SD*) cognitive performance as a function of location and exercise.

	**Memory (d’)**	**Digit Span Forwards**	**Digit Span Backwards**	**Trail Making A**	**Trail Making B**
Natural environment	1.86 (0.96)	92.45 (26.95)	43.45 (21.83)	19.39 (5.01)	39.86 (15.46)
Pre-Exercise	1.99 (0.94)	92.68 (29.23)	41.46 (21.85)	18.25 (4.99)	52.08 (17.91)
Post-Exercise	1.74 (0.98)	92.21 (24.99)	45.43 (22.03)	20.57 (4.84)	35.93 (12.58)
Indoor environment	1.86 (0.91)	84.98 (30.84)	42.05 (21.73)	21.34 (5.92)	39.49 (11.29)
Pre-Exercise	2.03 (0.81)	89.61 (34.86)	41.39 (18.58)	20.30 (5.70)	45.52 (13.13)
Post-Exercise	1.70 (0.91)	80.36 (26.04)	42.71 (24.82)	22.39 (6.04)	35.61 (8.00)
Pre-Exercise	2.01 (0.87)	91.14 (31.91)	41.43 (20.10)	19.27 (5.41)	47.71 (14.87)
Post-Exercise	1.72 (0.97)	86.29 (25.99)	44.07 (23.29)	21.49 (5.51)	35.77 (10.44)
Total	1.86 (0.93)	88.71 (29.07)	42.75 (21.70)	20.37 (5.55)	39.65 (13.23)

#### Digit Span

The product score for DSF was computed by multiplying the number correct (which could range from 0–16) by the length of the longest sequence accurately completed (which could range from 2–9), for a total score ranging from 0–144. There was a significant effect of environment, *F* (1, 27) = 4.12, *p* = 0.05, and η_*p*_^2^ = 0.14, such that participants scored higher in the natural environment than in the indoor environment. There was no significant effect of exercise [*F* (1, 27) = 2.73, *p* = 0.11, η_*p*_^2^ = 0.09], and no interaction [*F* (1, 27) = 0.98, *p* = 0.33, and η_*p*_^2^ = 0.04], see Table 2.

The product score for DSB was computed by multiplying the number correct (which could range from 0–14) by the length of the longest sequence accurately completed (which could range from 2–8), for a total score ranging from 0–112. There were no significant effects [Environment: *F* (1, 27) = 0.61, *p* = 0.44, and η_*p*_^2^ = 0.02; Exercise: *F* (1, 27) = 2.62, *p* = 0.12, and η_*p*_^2^ = 0.09; Interaction: *F* (1, 27) = 0.83, *p* = 0.37, and η_*p*_^2^ = 0.03), see Table 2.

#### Trail Making

For both A and B, versions 1 and 3 were always completed pre-exercise (counterbalanced for either the indoor or natural environment); similarly, versions 2 and 4 were always completed post-exercise, counterbalanced by environment. Thus, given the confound of version with exercise, it was important to determine if the 4 versions were comparable in difficulty (see Experiment 2). For TMTA, a repeated measures ANOVA revealed significant differences, *F* (3, 60) = 7.00, *p* < 0.001, and η_*p*_^2^ = 0.26. Post-hoc (LSD) tests revealed that TMTA version 1 (*M* = 17.01, *SE* = 0.83) and version 3 (*M* = 17.63, *SE* = 1.04) did not differ from each other, *p* = 0.56. Likewise, version 2 (*M* = 21.11, *SE* = 1.17), and version 4 (*M* = 20.53, *SE* = 0.99) did not differ from each other, *p* = 0.43. However, versions 1 and 3 differed significantly from versions 2 and 4 (*ps* < 0.05). For TMTB, a repeated measures ANOVA revealed significant differences, *F* (3, 60) = 2.69, *p* = 0.05, and η_*p*_^2^ = 0.19. Post-hoc (LSD) tests revealed that version 3 (*M* = 39.29, *SE* = 2.48) was significantly more difficult than version 1 (*M* = 35.30, *SE* = 1.90, and *p* = 0.05) and version 4 (*M* = 32.72, *SE* = 1.88, and *p* = 0.01). Versions 1, 2 (*M* = 35.67, *SE* = 2.36), and 4 did not significantly differ from each other, all *p*s > 0.05. Therefore, TMTB version 3 was excluded from analysis in the present study.

Thus, with the present sample, for TMTA, two *t*-tests were conducted separately to determine the effect of environment: (1) pre-exercise and (2) post-exercise. A paired-sample *t*-test revealed that, pre-exercise, those in the natural environment were marginally faster than those in the indoor environment, *t* (27) = 1.85, *p* = 0.08, Cohen’s *d* = 0.38. Similarly, post-exercise, those in the natural environment were marginally faster than those in the indoor environment, *t* (26) = 1.82, *p* = 0.08, Cohen’s *d* = 0.40, see Table 2.

With the present sample, time to complete TMTB (Versions 1, 2, and 4) was analyzed with the factors of environment and exercise in a normal identity generalized estimating equation. There was a main effect of exercise such that participants completed the task faster after exercise than before exercise, Wald χ2 (1, 83) = 20.16, *p* < 0.001, *QICC* = 11499.16. There was no effect of environment, Wald χ2 (1, 83) = 1.03, *p* = 0.31, *QICC* = 11499.16, and no interaction, Wald χ2 (1, 83) = 0.87, *p* = 0.35, *QICC* = 11499.16, see Table 2.

## Discussion

In contrast to the hypothesis, participants indicated greater happiness and positive affect in the indoor environment than in the natural environment. These results are in direct contrast to prior research (Bowler et al., 2010; McMahan and Estes, 2015), although other studies (Butryn and Furst, 2003; Kerr et al., 2006) have found no difference in mood as a result of running in an urban or natural environment. One possibility for the surprising difference in happiness and positive affect is that the natural environment location was not, in fact, restorative and may have even been perceived as dangerous. If this were the case, we would also expect stress and negative affect scores to differ across environments, but they were generally quite low and did not differ. This may indicate that there was not a great need for restoration in the first place, as participants were not currently undergoing stress or negative emotional experiences. However, the natural environment did result in improvement on cognitive tests requiring moderate amounts of attentional resources, indicating that there was room for at least some restoration to take place.

Solstice Canyon was deliberately chosen for its general popularity as an outdoor location, for its lack of crime, for the fact that it contains both green and blue elements, the ease of running on a hard-packed trail, and its general convenience to the University campus. Nevertheless, while natural environments are generally seen as restorative, such restoration is not universal. For instance, environments containing higher visibility (high prospect) and fewer hiding places (low refuge) are perceived as being more restorative, less dangerous, and result in higher attentiveness scores than low prospect, high refuge environments (Gatersleben and Andrews, 2013). In addition, for environments that are perceived as dangerous, being in nature with company (but not solitude) leads to restoration; solitude increased restoration only when danger was controlled for Staats and Hartig (2004). These findings demonstrate that the level of restoration from nature depends on the participants’ level of attention and fear, particularly for those who are alone, as was the case in this experiment. To examine the possibility that participants experienced fear, we compared the means from the PANAS item: the extent to which participants were currently feeling “Afraid.” The results showed no effect of environment, *F* (1, 27) = 0.00, *p* = 1.00, and η_*p*_^2^ = 0.00. Answers to an individual PANAS question (which are not typically examined in isolation), however, likely do not capture the full extent to which Solstice Canyon may have been perceived as fearful or dangerous, and do not touch at all on the perceived restoration of this environment. Thus, Experiment 2 explored the perceived restoration, fear, and danger of Solstice Canyon.

In regards to cognition, the effect of a natural environment was somewhat beneficial. There was no effect of environment on short or long term memory, but one working memory measure (DSF) and one measure of executive function (TMTA) were improved when in the natural environment compared to the indoor environment. It is worth noting that, contrary to Ohly et al. (2016), the versions requiring moderate attentional resources showed improvement from a natural environment, whereas versions requiring greater attentional resources (DSB, TMTB) showed no effect of environment. No cognitive measures showed worse performance for the natural environment compared to the indoor environment. These results, therefore, show partial support for ART, in that memory (which requires little attentional resources) was not affected by the environment, but tasks that require moderate attention (DSF, TMTA) showed improvements in a natural environment consistent with attentional restoration. This supports Stevenson et al.’s (2018) meta-analytic finding of disparate evidence for an attentional restoration mechanism. Two findings, however, suggest that factors other than attentional restoration may be at play. First, there was no effect of environment for the tasks with the greatest attentional demands, DSB and TMTB. Second, there were no significant interactions between environment and exercise, such that those in the natural environment did not show a larger restoration in affective or cognitive measures from pre to post exercise than those in the indoor environment. In the one marginal interaction, negative affect decreased more after exercise in the indoor environment than in the natural environment. While restoration cannot fully explain these results, neither can interest or motivation, as affective and cognitive effects of environment were not consistent across tasks, and neither can stress reduction, as stress was not affected by environment.

As hypothesized, exercise, regardless of environment, increased happiness and positive affect, and decreased stress and negative affect. In contrast to the hypothesis, exercise did not improve memory scores, and in fact resulted in marginally lower scores. This is surprising, given that long-term memory benefits are one of the more consistently supported effects of exercise (Lambourne and Tomporowski, 2010; Chang et al., 2012). Given the lack of consistency in regards to the effects of exercise on working memory, it is not surprising that exercise did not result in differences on DSF or DSB. When it could be assessed (TMTB), exercise did improve executive function. These results lend further clarity to the literature regarding differing effects of exercise on different cognitive tasks.

Overall, it appears that a natural environment has some beneficial effects on working memory and executive function tasks that require moderate amounts of attention, and no effects on tasks that require little attention. However, given the possibility that the natural environment location may not have been restorative, further investigation into this environment is needed before drawing definitive conclusions.

## Experiment 2

The purpose of Experiment 2 was to clarify the results from Experiment 1 in two ways. First, to compare the difficulty of the 4 different TMTA and 4 different TMTB versions, participants completed all 4 TMTA and all 4 TMTB versions from Experiment 1 in random order and in one sitting.

Second, in order to draw conclusions about the restorative effects of the natural environment used in Experiment 1, we measured the perceived restoration, perceived danger, and attentiveness to the natural location used in Experiment 1.

## Method

### Participants

Twenty-seven participants (17 women, 7 men; *M* age = 19.46, *SD* = 1.18) who did not participate in Experiment 1 were recruited.

### Materials

Participants completed an 11-item version of the Perceived Restorativeness Scale (PRS; Pasini et al., 2015), which was developed as a shorter version of the original PRS (Hartig et al., 1996). This 11-item scale measures the perceived restorative quality of natural environments with 4 groupings: Being Away (e.g., “Places like this are a refuge from nuisances”), Fascination (“e.g., In places like this my attention is drawn to many interesting things”), Coherence (e.g., “There is a clear order in the physical arrangement of places like this”), and Scope (e.g., “That place is large enough to allow exploration in many directions”). Each item was measured on a scale of 1 (*Strongly Agree)* to 7 (*Strongly Disagree)*.

Four questions (adapted from Gatersleben and Andrews, 2013) measured perceived danger (“I think I could come to harm during a walk through this place”), perceived fear (“I would be uneasy taking a walk through this place”), attentiveness (“I felt attentive to this place”), and behavior (“I would like to take a walk through this place”). Each item was measured on a scale of 1 (*Strongly Disagree)* to 7 (*Strongly Agree)*.

### Procedure

After giving informed consent, each participant completed the 4 TMTA and 4 TMTB in random order. All participants took the test in the same location (an indoor laboratory on the University’s campus) and did not exercise prior to the experiment. Next, participants viewed a slideshow that contained 32 images from Solstice Canyon Trail displayed for 6 seconds each; these images contained scenes that were either of the trail itself or of scenes that were easily visible from the trail. After viewing the slideshow, participants answered demographic questions and completed the PRS, danger, fear, attentiveness, and behavior items regarding the trail.

## Results

The data from 3 participants was discarded as excessive errors in the Trail Making portion of the task indicated they were not attending to the task. Trail Making results are described in Experiment 1.

To determine if the natural environment location was indeed restorative, the responses on the 11 PRS items were averaged (Hartig et al., 1996) and indicated that participants somewhat agreed that Solstice was restorative (*M* = 3.09, *SD* = 1.07). The mean was also separately calculated for the 3 Fascination items (*M* = 2.81, *SD* = 1.44), 3 Being Away items (*M* = 3.08, *SD* = 1.44), 3 Coherence items (*M* = 3.68, *SD* = 1.28), and 2 Scope (*M* = 2.67, *SD* = 1.52) items.

To examine the hypothesis that Solstice Canyon could induce fear, the mean was calculated for the two fear questions. The results suggested that participants disagreed that they would be uneasy about the location (*M* = 5.87, *SD* = 1.08) and somewhat disagreed that they could come to potential harm *(M* = 4.54, *SD* = 1.47). Further, participants agreed that they would like to take a walk through this place (*M* = 2.58, *SD* = 1.44). Lastly, participants somewhat agreed that they felt attentive to the location, (*M* = 2.96, *SD* = 1.00).

## General Discussion

The results indicated that Solstice did not induce fear and was not perceived as dangerous, that participants would like to walk there, and were attentive. Further, Solstice was perceived as somewhat restorative. Thus, the lack of greater positive affect and happiness for the natural environment was not due to fear, danger, inattention to the environment, or a lack of restoration. However, the lack of strong restorative scores on the PRS may explain why the expected affective benefits of the natural environment did not manifest, and perhaps why the cognitive benefits only manifested for tasks requiring only moderate amounts of attention—DSF and TMTA. If the environment was only somewhat restorative, it is possible that direct attention was only partially restored. Thus, tasks that require moderate amounts of attention may show the benefits of a small restoration, but tasks that require greater amounts of attention (DSB, TMTB) would not show significant improvement. One limitation to this interpretation, however, is that participants in Experiment 2, unlike Experiment 1, simply viewed the natural environment (consistent with Gatersleben and Andrews, 2013), rather than being present in the natural environment like in Experiment 1.

But why was the natural environment not more strongly restorative? An intriguing possibility is that while the natural environment contained all of the restorative elements, such as green and blue spaces, and low fear and danger, these types of natural environments are more commonly experienced by these participants, reducing its effect and the need for restoration. The University is located in Malibu, California, a location known not only for its highly urbanized landscape near Los Angeles, but also for its beautiful beaches and ocean views, rugged Santa Monica Mountains, and natural beauty with plentiful access to natural space. Compared to many other urban environments, green spaces are relatively accessible and numerous. If such experiences are frequent for most participants, then perhaps a natural environment would not be as strongly restorative or have as strong affective benefits. Further, the results from this participant population, who have relatively easy and frequent access to natural environments, may not generalize to populations for whom natural environments are far less accessible and/or safe. Future research should consider the inclusion of a sample with greater diversity in access to nature.

This possibility is consistent with hedonic adaptation, wherein the emotional effects of a stimulus become weakened with repeated experience of that stimulus (Frederick and Loewenstein, 1999). Researchers have suggested that this adaptation and subsequent weakening of affective reactions is due to a reduction in attention to that stimulus; novel, self-relevant stimuli that once captured attention no longer do so (Wilson and Gilbert, 2008). However, our results indicated that participants in Experiment 2 were attentive to the environment. This “variety is the spice of life” explanation is not consistent with research showing that more time spent in nature is linked to greater positive affect [an effect partially accounted for by the quality of the nature experience (i.e., fascination; Sato and Connor, 2013)] and that increased exposure or availability of natural environments is related to many benefits, such as better mental health (Alcock et al., 2014), less stress, depression, and anxiety (Thompson et al., 2012; Beyer et al., 2014), and greater occupational well-being (Hyvönen et al., 2018). Future research should investigate the possibility that with repeated exposure, each individual exposure results in smaller *immediate* affective benefits due to hedonic adaptation, but that greater cumulative exposure results in larger long-term affective benefits. Even if the effect of environment on affect is smaller for those who experience natural environments regularly, our results show that such environments can still benefit cognition. Future studies should investigate the possibility that our findings of increased performance on DSF and TMTA would be more robust for those who are unused to natural environments, perhaps extending to DSB and TMTB.

An additional possibility is that there may have been an incongruency between the natural environment and experiment expectations. In other words, participants may have expected and been intrinsically motivated for exploration and relaxation in a natural environment, but were instead met with a prescribed running route and with cognitively demanding tasks. Specifically, participants lacked agency and autonomy to choose how they spent their time in the natural environment (e.g., Ryan and Deci, 2000; Andringa et al., 2013) and were asked to perform tasks that were incongruent with the environment. Such incongruency could explain lower positive affect and happiness. Conversely, the indoor environment likely fostered a higher congruency between expectations and demands; many of the participants were students or professors of the University where the indoor environment was located and likely associated that location with more prescribed and cognitively demanding tasks.

The environments also differ in ways other than simple exposure to nature. In Experiment 1, the indoor location, despite being a more impoverished and controlled environment, approximates situations (e.g., an office, school) where the type of cognitive tests used in this and similar research are likely to be relevant. The natural environment, however, is richer and less controlled. In Experiment 2, the natural environment was viewed from a more controlled laboratory setting. Future research into the effects of natural environments should take into account and further explore how laboratory environments differ from natural environments, and how these differences may contribute to different motivations and behaviors.

In accordance with our first aim, using a within-subjects design and non-fatigued control groups, we found some support for ART, with tasks that required moderate attentional resources showing the greatest benefit of a natural environment, and tasks showing little attentional demand (memory) showing no difference. However, the lack of improvement in tasks requiring the most amount of direct attention, and the lack of an interaction with exercise, suggests that ART cannot fully explain cognitive performance in natural and indoor environments. With the pre-exercise measures serving as a non-fatigued control for both environments, post-exercise (fatigued) benefits for executive function in both environments suggest that either attentional restoration (stemming purely from the environment) is not the explanation for improved post-exercise performance, or that participants were not in fact suffering from depleted direct attentional resources in the first place.

Regarding our second aim, the effects of exercise were generally, with the exception of memory, consistent with our hypotheses—improved affect, no effect on working memory, and some benefit to executive function. While the picture is becoming clearer regarding exercise effects on working memory and executive function, further research is still needed to tease out the factors that might lead to benefits in some circumstances with some tasks, and no such benefits in others. In conclusion, exercise and environment both appear to improve cognitive performance across different tasks, while affective benefits may depend on other factors, such as how regularly one experiences natural environments. These findings lend further support to the need to provide and maintain accessible restorative natural environments. However, while understanding the environment is critical to understanding affective and cognitive behavior, it is important to remember that natural environments are not always universally beneficial.

## Data Availability Statement

The raw data supporting the conclusions of this article will be made available by the authors, without undue reservation.

## Ethics Statement

The studies involving human participants were reviewed and approved by Institutional Review Board of Pepperdine University. The patients/participants provided their written informed consent to participate in this study.

## Author Contributions

JT developed the research idea, methodology, conducted all data analyses, and wrote the initial draft of the manuscript. SA was an active contributor to methodology, collected all data, and provided substantial and meaningful contributions to interpretation of results and writing of the manuscript. Both authors contributed to the article and approved the submitted version.

## Conflict of Interest

The authors declare that the research was conducted in the absence of any commercial or financial relationships that could be construed as a potential conflict of interest.

## References

[B1] Alcock I. White M. P. Wheeler B. W. Fleming L. E. Depledge M. H. (2014). Longitudinal effects on mental health of moving to greener and less green urban areas. *Environ. Sci. Technol.* 48 1247–1255. 10.1021/es403688w 24320055

[B2] Andringa T. C. van den Bosch K. A. M. Vlaskamp C. (2013). Learning autonomy in two or three steps: linking open-ended development, authority, and agency to motivation. *Front. Psychol.* 4:766 10.3389/fpsyg.2013.00766 PMC3805057 24155734

[B3] Berman M. G. Jonides J. Kaplan S. (2008). The cognitive benefits of interacting with Nature. *Psychol. Sci.* 19 1207–1212. 10.1111/j.1467-9280.2008.02225.x 19121124

[B4] Berto R. (2005). Exposure to restorative environments helps restore attentional capacity. *J. Environ. Psychol.* 25 249–259. 10.1016/j.jenvp.2005.07.001

[B5] Beyer K. M. Kaltenbach A. Szabo A. Bogar S. Nieto F. J. Malecki K. M. (2014). Exposure to neighborhood green space and mental health: evidence from the survey of the health of Wisconsin. *Int. J. Environ. Res. Public Health* 11 3453–3472. 10.3390/ijerph110303453 24662966 PMC3987044

[B6] Bodin M. Hartig T. (2003). Does the outdoor environment matter for psychological restoration gained through running? *Psychol. Sport Exer.* 4 141–153. 10.1016/S1469-0292(01)00038-3

[B7] Borg G. A. (1982). Psychophysical bases of perceived exertion. *Med. Sci. Sports Exer.* 14 377–381. 10.1249/00005768-198205000-00012 7154893

[B8] Bowie C. R. Harvey P. D. (2006). Administration and interpretation of the Trail Making Test. *Nat. Protoc.* 1 2277–2281. 10.1038/nprot.2006.390 17406468

[B9] Bowler D. E. Buyung-Ali L. M. Knight T. M. Pullin A. S. (2010). A systematic review of evidence for the added benefits to health of exposure to natural environments. *BMC Public Health* 10:456 10.1186/1471-2458-10-456 PMC2924288 20684754

[B10] Butryn T. M. Furst D. M. (2003). The effects of park and urban settings on the moods and cognitive strategies of female runners. *J. Sport Behav.* 26 335–355.

[B11] Chang Y. K. Labban J. D. Gapin J. I. Etnier J. L. (2012). The effects of acute exercise on cognitive performance: a meta-analysis. *Brain Res.* 1453 87–101. 10.1016/j.brainres.2012.02.068 22480735

[B12] Colcombe S. Kramer A. F. (2003). Fitness effects on the cognitive function of older adults: a meta-analytic study. *Psychol. Sci.* 14 125–130. 12661673 10.1111/1467-9280.t01-1-01430

[B13] Coon J. T. Boddy K. Stein K. Whear R. Barton J. Depledge M. H. (2011). Does participating in physical activity in outdoor natural environments have a greater effect on physical and mental wellbeing than physical activity indoors? A systematic review. *Environ. Sci. Technol.* 45 1761–1772. 10.1021/es102947 21291246

[B14] Farina N. Rusted J. Tabet N. (2014). The effect of exercise interventions on cognitive outcome in Alzheimer’s disease: a systematic review. *Int. Psychogeriatr.* 26 9–18. 10.1017/S1041610213001385 23962667

[B15] Finlay J. Franke T. MacKay H. Sims-Gould J. (2015). Therapeutic landscapes and wellbeing in later life: impacts of blue and green spaces for older adults. *Health Place* 34 97–106. 10.1016/j.healthplace.2015.05.001 25982704

[B16] Frederick S. Loewenstein G. (1999). “Hedonic adaptation,” in *Well-Being: The Foundations of Hedonic Psychology*, eds Kahneman D. Diener E. Schwarz N. (New York, NY: Russell Sage Foundation), 302–329.

[B17] Gamble K. R. Howard J. H. Jr. Howard D. V. (2014). Not just scenery: Viewing nature pictures improves executive attention in older adults. *Exp. Aging Res.* 40 513–530. 10.1080/0361073X.2014.956618 25321942 PMC4929355

[B18] Gascon M. Triguero-Mas M. Martínez D. Dadvand P. Forns J. Plasència A. (2015). Mental health benefits of long-term exposure to residential green and blue spaces: a systematic review. *Int. J. Environ. Res. Public Health* 12 4354–4379. 10.3390/ijerph120404354 25913182 PMC4410252

[B19] Gatersleben B. Andrews M. (2013). When walking in nature is not restorative–The role of prospect and refuge. *Health Place* 20 91–101. 10.1016/j.healthplace.2013.01.001 23399852

[B20] Gidlow C. J. Jones M. V. Hurst G. Masterson D. Clark-Carter D. Tarvainen M. P. (2016). Where to put your best foot forward: psycho-physiological responses to walking in natural and urban environments. *J. Environ. Psychol.* 45 22–29. 10.1016/j.jenvp.2015.11.003

[B21] Hartig T. Korpela K. M. Evans G. W. Gärling T. (1996). “Validation of a measure of perceived environmental restorativeness,” in *Göteborg Psychological Reports*, Vol. 26 (Göteborg: Göteborg University), 1–64.

[B22] Hötting K. Schauenburg G. Röder B. (2012). Long-term effects of physical exercise on verbal learning and memory in middle-aged adults: results of a one-year follow-up study. *Brain Sci.* 2 332–346. 10.3390/brainsci2030332 24961197 PMC4061798

[B23] Hyvönen K. Törnroos K. Salonen K. Korpela K. Feldt T. Kinnunen U. (2018). Profiles of nature exposure and outdoor activities associated with occupational well-being among employees. *Front. Psychol.* 9:754 10.3389/fpsyg.2018.00754 PMC5968374 29867699

[B24] Joye Y. Dewitte S. (2018). Nature’s broken path to restoration. A critical look at attention restoration theory. *J. Environ. Psychol.* 59 1–8. 10.1016/j.jenvp.2018.08.006

[B25] Kaplan S. (1995). The restorative benefits of nature: toward an integrative framework. *J. Environ. Psychol.* 15 169–182. 10.1016/0272-4944(95)90001-2

[B26] Kaplan S. Berman M. G. (2010). Directed attention as a common resource for executive functioning and self-regulation. *Perspect. Psychol. Sci.* 5 43–57. 10.1177/1745691609356784 26162062

[B27] Kerr J. H. Fujiyama H. Sugano A. Okamura T. Chang M. Onouha F. (2006). Psychological responses to exercising in laboratory and natural environments. *Psychol. Sport Exer.* 7 345–359. 10.1016/j.psychsport.2005.09.002

[B28] Kessels R. P. C. van Zandvoort M. J. E. Postma A. Kappelle L. J. De Haan E. H. F. (2000). The corsi block-tapping task: standardization and normative data. *Appl. Neuropsychol.* 7 252–258. 10.1207/S15324826AN0704_8 11296689

[B29] Lambourne K. Tomporowski P. (2010). The effect of exercise-induced arousal on cognitive task performance: a meta-regression analysis. *Brain Res.* 1341 12–24. 10.1016/j.brainres.2010.03.091 20381468

[B30] Loprinzi P. D. Frith E. Edwards M. K. Sng E. Ashpole N. (2018). The effects of exercise on memory function among young to middle-aged adults: systematic review and recommendations for future research. *Am. J. Health Promot.* 32 691–704. 10.1177/0890117117737409 29108442

[B31] McMahan E. A. Estes D. (2015). The effect of contact with natural environments on positive and negative affect: a meta-analysis. *J. Posit. Psychol.* 10 507–519. 10.1080/17439760.2014.994224

[B32] Naderi A. Shaabani F. Esmaeili A. Salman Z. Borella E. Degens H. (2019). Effects of low and moderate acute resistance exercise on executive function in community-living older adults. *Sport Exer. Perform. Psychol.* 8 106–122. 10.1037/spy0000135

[B33] Ohly H. White M. P. Wheeler B. W. Bethel A. Ukoumunne O. C. Nikolaou V. (2016). Attention restoration theory: a systematic review of the attention restoration potential of exposure to natural environments. *J. Toxicol. Environ. Health Part B* 19 305–343. 10.1080/10937404.2016.1196155 27668460

[B34] Öhman H. Savikko N. Strandberg T. E. Pitkälä K. H. (2014). Effect of physical exercise on cognitive performance in older adults with mild cognitive impairment or dementia: a systematic review. *Dement. Geriatr. Cogn. Disord.* 38 347–365. 10.1159/000365388 25171577

[B35] Pasini M. Berto R. Brondino M. Hall R. Ortner C. (2015). How to measure the restorative quality of environments: The PRS-11. *Proc. Soc. Behav. Sci.* 159 293–297. 10.1016/j.sbspro.2014.12.375

[B36] Potter D. Keeling D. (2005). Effects of moderate exercise and circadian rhythms on human memory. *J. Sport Exerc. Psychol.* 27 117–125. 10.1123/jsep.27.1.117

[B37] Pretty J. Peacock J. Hine R. Sellens M. South N. Griffin M. (2007). Green exercise in the UK countryside: effects on health and psychological well-being, and implications for policy and planning. *J. Environ. Plan. Manag.* 50 211–231. 10.1080/09640560601156466

[B38] Rey A. (1964). *L’examen Clinique en Psychologie.* Paris: Presses universitaires de France.

[B39] Rider N. D. Bodner G. E. (2016). Does taking a walk in nature enhance long-term memory? *Ecopsychology* 8 27–34. 10.1089/eco.2015.0042

[B40] Rogerson M. Barton J. (2015). Effects of the visual exercise environments on cognitive directed attention, energy expenditure and perceived exertion. *Int. J. Environ. Res. Public Health* 12 7321–7336. 10.3390/ijerph120707321 26133125 PMC4515658

[B41] Ryan R. M. Deci E. L. (2000). Intrinsic and extrinsic motivations: classic definitions and new directions. *Contemp. Educ. Psychol.* 25 54–67. 10.1006/ceps.1999.1020 10620381

[B42] Sato I. Connor T. S. (2013). The quality of time in nature: how fascination explains and enhances the relationship between nature experiences and daily affect. *Ecopsychology* 5 197–204. 10.1089/eco.2013.0026

[B43] Schertz K. E. Berman M. G. (2019). Understanding nature and its cognitive benefits. *Curr. Direct. Psychol. Sci.* 28 496–502. 10.1177/0963721419854100

[B44] Staats H. Hartig T. (2004). Alone or with a friend: a social context for psychological restoration and environmental preferences. *J. Environ. Psychol.* 24 199–211. 10.1016/j.jenvp.2003.12.005

[B45] Stevenson M. P. Schilhab T. Bentsen P. (2018). Attention restoration theory II: a systematic review to clarify attention processes affected by exposure to natural environments. *J. Toxicol. Environ. Health Part B* 21 227–268. 10.1080/10937404.2018.1505571 30130463

[B46] Tan B. W. Pooley J. A. Speelman C. P. (2016). A meta-analytic review of the efficacy of physical exercise interventions on cognition in individuals with autism spectrum disorder and ADHD. *J. Autism Dev. Disord.* 46 3126–3143. 10.1007/s10803-016-2854-x 27412579

[B47] Taylor A. F. Kuo F. E. (2009). Children with attention deficits concentrate better after a walk in the park. *J. Attent. Disord.* 12 402–409. 10.1177/1087054708323000 18725656

[B48] Thompson C. W. Roe J. Aspinall P. Mitchell R. Clow A. Miller D. (2012). More green space is linked to less stress in deprived communities: evidence from salivary cortisol patterns. *Landsc. Urban Plan.* 105 221–229. 10.1016/j.landurbplan.2011.12.015

[B49] Tomporowski P. D. Lambourne K. Okumura M. S. (2011). Physical activity interventions and children’s mental function: an introduction and overview. *Prevent. Med.* 52 S3–S9. 10.1016/j.ypmed.2011.01.028 PMC3160636 21420981

[B50] Ulrich R. S. Simons R. F. Losito B. D. Fiorito E. Miles M. A. Zelson M. (1991). Stress recovery during exposure to natural and urban environments. *J. Environ. Psychol.* 11 201–230. 10.1016/S0272-4944(05)80184-7

[B51] Watson D. Clark L. A. Tellegan A. (1988). Development and validation of brief measures of positive and negative affect: the PANAS scales. *J. Pers. Soc. Psychol.* 54 106 10.1037/0022-3514.54.6.1063 3397865

[B52] White M. Smith A. Humphyres K. Pahl S. Snelling D. Depledge M. (2010). Blue space: the importance of water for preference, affect, and restorativeness ratings of natural and built scenes. *J. Environ. Psychol.* 30 482–493. 10.1016/j.jenvp.2010.04.004

[B53] Wilson T. D. Gilbert D. T. (2008). Explaining away: a model of affective adaptation. *Perspect. Psychol. Sci.* 3 370–386. 10.1111/j.1745-6924.2008.00085.x 26158955

